# Healthcare Professionals’ Attitudes toward Rapid Whole Genome Sequencing in Pediatric Acute Care

**DOI:** 10.3390/children9030357

**Published:** 2022-03-04

**Authors:** Linda S. Franck, Andrea Scheurer-Monaghan, Caleb P. Bupp, Joseph D. Fakhoury, Thomas J. Hoffmann, Manasi Deshpandey, Madison Arenchild, David P. Dimmock

**Affiliations:** 1 Department of Family Health Care Nursing, University of California, San Francisco, CA 94143, USA; manasi.deshpandey@ucsf.edu; 2 Neonatal Intensive Care, Bronson Children’s Hospital, Kalamazoo, MI 49007, USA; scheurea@bronsonhg.org; 3 Department of Pediatric and Adolescent Medicine, Western Michigan University Homer Stryker School of Medicine, Kalamazoo, MI 49007, USA; fakhourj@bronsonhg.org; 4 Medical Genetics, Spectrum Health Helen DeVos Children’s Hospital, Grand Rapids, MI 49503, USA; caleb.bupp@spectrumhealth.org; 5 Department of Pediatrics and Human Medicine, College of Human Medicine, Michigan State University, Grand Rapids, MI 49503, USA; 6 Pediatric Hospital Medicine, Bronson Children’s Hospital, Kalamazoo, MI 49007, USA; 7 Department of Epidemiology and Biostatistics, University of California, San Francisco, CA 94158, USA; thomas.hoffmann@ucsf.edu; 8 Rady Children’s Institute for Genomic Medicine, San Diego, CA 92123, USA; marenchild@rchsd.org (M.A.); dpdimmock@gmail.com (D.P.D.)

**Keywords:** genetics, genomics, pediatrics, hospital medicine, medical technology and advancement

## Abstract

We aimed to characterize knowledge and attitudes about rapid whole genome sequencing (rWGS) implementation of a broad constituency of healthcare professionals at hospitals participating in a statewide initiative to implement rWGS for hospitalized neonates and children up to 18 years of age meeting clinical criteria for testing. We surveyed 307 healthcare professionals from eight hospitals about their knowledge and attitudes regarding rWGS. We examined survey internal reliability using exploratory factor analysis and associations between respondent characteristics and attitudes toward rWGS with linear regression. We thematically analyzed free-text responses. Views about rWGS implementation in respondents’ own setting and respondents’ personal capability to implement rWGS were generally neutral (M = 3.44 (SD = 0.74); M = 3.30 (SD = 0.85), respectively). Views about the potential for rWGS in clinical practice were overall positive (M = 4.12 (SD = 0.57)). The degree of positivity of attitudes about rWGS was strongly influenced by perceived knowledge, clinical or non-clinical role, concerns about future insurance coverage for rWGS as a first-tier test, and future adverse impact of genomics health information on patients or families. We identified several actionable factors influencing attitudes toward rWGS of pediatric healthcare professionals. Expanded education and ongoing implementation research are needed for the full potential of rWGS in pediatrics to be realized.

## 1. Introduction

Research and quality improvement studies of whole genome sequencing across multiple settings and countries consistently report higher diagnostic yield than standard genetic testing. Rapid genome-wide sequencing, also commonly known as rapid whole genome sequencing (rWGS), with results reported within 2–3 days, is a recent development in precision medicine [1]. With fast turn-around times, rWGS is poised to become a first-tier test in pediatric critical and acute care settings [1,2,3,4,5,6,7,8,9,10,11,12]. As cost of testing steadily falls, rWGS is becoming cost-effective when ordered early in the diagnostic evaluation of suspected genetic conditions or conditions of unclear or unknown etiologies [12,13,14,15].

Effective clinical implementation of new technology includes consideration of factors beyond the efficacy and cost of the technology. The knowledge and attitudes of direct care providers, support staff, and hospital leaders along with the perceptions of patients, families and the public are extremely influential in determining the degree and speed of adoption (or non-adoption) of any new technology [16]. Little is known about implementation in clinical practice of rWGS in pediatric acute care.

In our recent qualitative analysis of a protocol-driven five-hospital implementation of rWGS for critically ill infants, we described common themes, of which the role of clinical champions, provider education and perceptions about rWGS were prominent and variable in approach across the sites [17]. In the present study, we sought to better understand the knowledge and attitudes about rWGS implementation of a broad constituency of healthcare professionals involved in a state-wide initiative to introduce rWGS as a first-tier test for children meeting eligibility criteria. A better understanding of the views of key stakeholders in early-stage implementation can inform interventions to more effectively address new technology adoption across pediatric healthcare settings.

## 2. Materials and Methods

### 2.1. Project Start-up and Participants

In 2020, Michigan launched Project Baby Deer, a statewide initiative with the goal of offering rWGS to hospitalized neonates and children up to 18 years of age who met clinical criteria for testing, regardless of location or type of insurance [18]. All children’s hospitals in the state were invited to participate. The Michigan Hospital Association provided interested sites with guidance to help streamline institutional approvals and laboratory service agreements. Physician project champions were identified for each site. Onboarding at each site included a kick-off meeting facilitated by the Rady Institute for Genomic Medicine during which the objectives of the quality improvement project were described, and project champions and their teams were informed about how to identify cases and procedures for obtaining consent, ordering rWGS, and submitting clinical and phenotypic [17]. Monthly case review sessions were provided virtually throughout the project. Additional communication, in-service education or other on-the-job training activities were determined individually for each site by the clinical site champions and the local teams.

As of March 2021, nine hospital sites from five different health systems were signed on to the project. The designated clinical champion from each site agreed to share the invitation to participate in the voluntary online survey via email distribution lists. All members of the hospital staff with potential for involvement in the implementation of rWGS at each site were invited to participate, including but not limited to neonatologists, pediatric intensivists, pediatric hospitalists, subspecialists, fellows, residents, nurse practitioners, physician assistants, division chiefs/medical directors, geneticists, genetic counselors, nurse managers, nurses, social workers, laboratory directors, laboratory staff, and hospital administrators. The study was reviewed and deemed exempt from review by the University of California, San Francisco Committee on Human Research. Participants did not receive any compensation for participation.

### 2.2. Survey

We designed a 44-item survey with eight demographic items, four items about experience with rWGS in practice, two items about genomics education and perceived knowledge, five items about use of rWGS resources, 16 items about views on rWGS implementation as part of Project Baby Deer, and seven items about the potential for wider adoption of rWGS in clinical practice and personal intentions to use rWGS in one’s own practice. The items exploring experience with rWGS and Project Baby Deer implementation were informed by our prior qualitative research [17], and views on rWGS implementation and wider adoption of rWGS were adapted from existing scales [19,20,21]. Two open-ended questions were included for participants to elaborate on their views of rWGS implementation at their site or regarding wider adoption of rWGS in the care of acutely ill infants and children. The survey is available at: https://bit.ly/3HxUNIA (accessed on 23 February 2022).

### 2.3. Statistical Analysis

We first described the respondents’ demographic and practice characteristics, self-reported prior genetics education, and experience with rWGS using means and standard deviations, or counts and frequencies. Divergent stacked bar plots were created in R v4.1 [22] using HH v3.1.43 [23]. We conducted multiple imputation to appropriately handle missing data [24], using 100 imputations. For exploratory factor analysis, we calculated the polychoric correlation matrix [25] of the ordinal 5-point Likert scale questions on each imputation and then averaged them [26].

We conducted exploratory factor analysis to group questionnaire items into a smaller number of latent variables. Items with correlation ≥0.3 with other items were included. The Kaiser–Meyer–Olkin test was 0.91, indicating our data were well-suited for exploratory factor analysis. Solutions for 3–6 factors were each examined using oblique and orthogonal rotations of the factor loading matrix; the three-factor solution was preferred because of the leveling off of eigenvalues on the scree plot after three factors, having at least two items per factor, an insufficient number of primary loadings and difficulty of interpreting the fourth factor, and subsequent factors. To estimate the independent associations of the identified factors with terms for respondent demographics, knowledge, and concerns about genomics testing, we first fit a linear regression of each factor with each term. We then fit a stepwise regression based on Bayesian information criteria to build a multivariate model. These regression models were fit using a standard multiple imputation approach, where the regression model is fit to each of the multiply imputed datasets, and then these results are appropriately pooled together [24].

We examined the free-text responses to the open-ended questions using summative thematic analysis [27], identifying key words, tabulating frequencies, and identifying exemplar quotes for themes relating to views on rWGS implementation and potential impact on patient care.

## 3. Results

### 3.1. Participant Characteristics

Demographics for the 307 respondents are shown in Table 1. One of the nine invited sites declined to participate. The response rate for the remaining eight sites was 34%. Forty-five percent (*n* = 148) of respondents identified as professionals who had selection and ordering of genetic tests within their clinical roles (i.e., attending physicians, geneticists or genetic counselors and nurse practitioners). Another 46% (*n* = 142) had other direct patient care or patient and family support roles such as nurses, respiratory therapists or social workers. The remaining 9% (*n* = 27) had other non-direct patient care roles such as laboratory personnel or hospital administrators. Respondents from the two hospitals that were the first to implement rWGS comprised 42% (*n* = 129) and 35% (*n* = 107) of the sample (early adopters), with each of the remaining six hospitals representing 2–6% of the sample (later adopters). The mean years in practice ranged from less than one year to over 50 years, with medical residents and some nurses having fewer years of experience and hospital administrators, nurse managers/directors and some attending physicians having more years of experience. In the previous 6 months, 52% (*n* = 161) of respondents had been involved in the care of an inpatient child for whom rWGS was ordered, with 45% (*n* = 138) caring for patients or families who were then referred for genetic counseling because of rWGS results and 25% (*n* = 76) having had direct conversations with families about rWGS testing or diagnosed disorders. The frequency of these responses indicated exposure to rWGS did not differ by site.

### 3.2. rWGS Education

Overall, 75% (*n* = 231) of the sample had received one or more types of genetics or genomics training (median = 2 trainings; IQR: 1,4). Table 2 shows the types of genetics/genomics education respondents reported receiving. The three most common were on-the-job training (53%), genetics course in initial professional training (42%) and hospital-supported training (37%). Respondents from the later adopter sites more often reported receiving genetics/genomics education versus respondents from the early adopter sites (*p* < 0.001). However, there were no differences in type of genetics/genomics education received by site.

The most common self-rated level of knowledge of rWGS for acutely ill children was “a little” (34%; *n* = 103), followed by “none” (29%; *n* = 90). Few respondents rated themselves as having “expert” (1.3%, *n* = 4) knowledge about rWGS. There were significant differences between position type and genetics education, with 98% (*n* = 124) of providers reporting genetics education and only 53% (*n* = 69) of direct care nurses having any genetics education (*p* < 0.001). Only 64% (16/25) geneticists and genetics counselors self-rated their knowledge as “a lot” or “expert”.

### 3.3. Factor Analysis—rWGS Attitudes Scale

A total of 18 of the 23 items were suitable for factor analysis (correlation ≥ 0.3 with another item). The five items dropped related to concerns about insurance coverage and implications of genomics testing for diverse patients. Three factors were identified, with six items related to perceived personal capability to incorporate rWGS into one’s clinical practice, six items related to beliefs about the potential of rWGS to enhance current or future patient care and intention to implement, and six items about the perceived quality of rWGS implementation in one’s unit or hospital (Table 3). These three factors explained 55% of the variance (19%, 19%, and 17% for each factor, respectively). The Cronbach’s α reliability coefficient was 0.92 overall, and 0.87, 0.87 and 0.85 for each of the factors, respectively.

### 3.4. Views about rWGS Implementation and Implications for Clinical Practice

**Figure 1:**
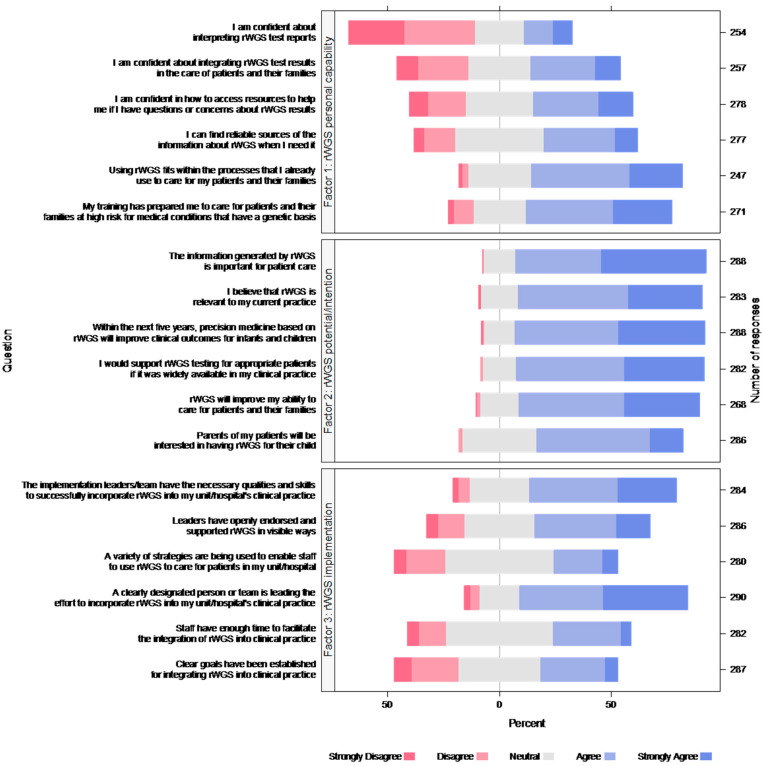
Respondent views about rWGS. Each color bar represents percent of responses.

Respondents’ views about rWGS implementation within their unit/hospital as a part of Project Baby Deer were generally neutral (subscale M = 3.44; SD = 0.74). Similarly, views about one’s personal capability to implement rWGS were generally neutral (subscale M = 3.30; SD = 0.85), except for confidence about interpreting rWGS test reports, which respondents rated less positively (M = 2.49; SD = 1.24). Views about rWGS in future clinical practice beyond Project Baby Deer were overall positive (M = 4.12; SD = 0.57) (Figure 1).

### 3.5. Variables Associated with rWGS Factor and Total Attitudes Scores

We first fit a univariate regression model for crude associations of respondent demographics, role, site, knowledge level, and the five questions regarding concerns about genomics testing with each of the factor scores and total attitudes score. The univariate regression model showed nominal (*p* < 0.05) associations for many of the variables with the factors (personal capability, potential/intention, implementation), and generally, if a term was associated with one factor, it was associated with all factors and total score (Table S1).

The stepwise multivariate regression model utilizing these same terms and, for each of the factor scores (personal capability, potential/intention, implementation), explained 43%, 44%, and 32% of the variability of the factor scores, respectively, and 51% of the variability in the total score. The variables rWGS self-rated knowledge and a positive expectation of future insurance coverage for rWGS were significant in the final model for all three factors and the total score, type of unit (critical care vs. non-critical care) was significant only in the personal capability factor final model, concerns about racial disparities in future rWGS use and concerns about possible negative long-term effect of genomic testing for patients/families were significant only in the potential/intention factor final model, and site 2 and position type (direct clinical care vs. not) were significant only in the implementation factor final model. Self-rated knowledge, position type and confidence about future insurance coverage for rWGS were significant in the final model for all three factors and the total score (Table 4).

### 3.6. Respondent Comments about rWGS Implementation and Wider Adoption

Approximately one-third (*n* = 110) of the respondents provided 172 free-text responses to the two open-ended questions inviting respondents to elaborate on their views regarding rWGS implementation and wider adoption. The main themes identified were implementation, potential for current and future patient care, and concerns (Table 5). Most comments (38.5%; *n* = 66) expressed respondents’ general excitement for rWGS and its actual (or potential) to improve diagnostic accuracy and speed, leading to more effective treatment. Some of these comments expressed qualified enthusiasm for wider adoptions related to the barrier of limited insurance coverage or desire for more cost/utility data. Another 34.5% (*n* = 59) of comments focused on aspects of implementation of the rWGS in the respondents’ unit or hospital. About one-third of these comments expressed satisfaction with the implementation and others indicated limited experience but interest in learning more. Some respondents commented about the genetics service having a primary role in rWGS implementation. Sixteen percent (*n* = 28) of comments indicated lack of awareness or irrelevance of the rWGS to the respondents’ clinical practice. Eleven percent (*n* = 19) of comments expressed concerns or cautions about wider adoption of rWGS, including need for clear and specific criteria to prevent misuse, need for more training of the patient care team, concerns about availability of genetics team support, and potential future risks of genomics information for patients and their families. This section may be divided by subheadings. It should provide a concise and precise description of the experimental results, their interpretation, as well as the experimental conclusions that can be drawn.

## 4. Discussion

Our survey is the largest study of health professionals’ views of rWGS to date and included a broad constituency of professionals involved in the early stages of a state-wide initiative to introduce rWGS as a first-tier test for children meeting eligibility criteria. Overall, most respondents agreed that rWGS is an important diagnostic tool relevant to their practice and likely to improve care. Most indicated they would support rWGS testing for appropriate patients if available to them in their practice. Although respondent opinion on patient interest in rWGS cannot be substituted for directly surveying patients or parents themselves, most respondents felt that parents would be interested in rWGS if offered.

Previous studies evaluated hypothetical situations [28], trials [29], were retrospective [17], or were limited to intensivist physicians, genetics and laboratory professionals [30,31,32]. Consistent with other international studies [33], we discovered that attitudes were generally favorable about the implementation process, the potential for immediate and long-term benefit for patients, and intention to incorporate rWGS into practice. However, the degree of positivity was strongly influenced by perceived knowledge, clinical or non-clinical role, degree of confidence about future insurance coverage for rWGS as a first-tier test, and concern about future adverse impact of genomics health information on patients or families. Our findings support and extend findings from a previous survey of physicians directly involved in ordering rWGS on critically ill children with conditions of unknown etiology that found ordering physicians perceived high clinical utility and low likelihood of harm with first-tier rWGS, irrespective of the outcome of the test [1].

Our study is the first to explore the internal reliability of a survey of pediatric health professional’s views about rWGS as a first-tier diagnostic in pediatric acute and critical care practice and to examine relationships between personal and organizational characteristics on their views. We encourage adoption of the survey in clinical practice and research to build knowledge about factors influencing the adoption of rWGS or other new genomic diagnostics in pediatrics and to evaluate the impact of interventions to address knowledge and concerns.

Our previous study of rWGS implementation identified five key implementation themes: the importance of rWGS champions, educational needs and strategies, negotiating decision-making roles, workflows, and perceptions about rWGS [17]. In our current study, each site had a designated clinical champion. Consistent with our previous findings, the site clinical champion was most often a physician provider and was perceived to be equally effective in the role whether from genetics, neonatology, intensivist, hospitalists or other specialty. Despite positive perceptions overall, and consistent with other studies [33], respondents reported a lack of confidence in interpreting rWGS results, accessing resources related to testing, or integrating rWGS results into patient care plans. Some respondents had limited exposure to rWGS in clinical care, as the project is still at the early stages. Moreover, the findings suggest that additional efforts to engage nurses are needed.

Precision medicine and genomics is a new frontier in pediatrics and is yet to be included in standard education or exams. If the potential of rWGS and other next generation genomic diagnostics and therapeutics is to be fully realized in pediatrics, research is concurrently needed to evaluate the most effective strategies for efficiently educating the current clinical workforce and future workforce about precision genomic medicine. Policy statements and guidelines from medical societies on genomic testing in neonatology and pediatrics would empower champions and aid in widespread adoption.

Ongoing education and implementation efforts should focus on understanding, interpreting and communicating rWGS results and aim to include the entire patient care team. Resources to support staff with implementation of a test that is not used daily need to be accessible and easy to use. Improving access to resources related to rWGS and rare diseases, as well as utilizing telemedicine support for specialist support in interpretation of results related to rare diseases, would likely help increase clinician confidence and capability.

As we found in our previous study [17], a common issue arising in implementation of rWGS is the need to negotiate roles and responsibilities for initiating rWGS and interpreting and communicating results. This recurring theme was reflected in the comments provided by respondents, with some expressing strong views about the need for active involvement or oversight of rWGS by the genetics service, whereas others expressing equally strong views that rWGS should be a first-tier test managed by front-line providers. Both approaches have strengths and challenges, and the optimal approach is likely to be highly context dependent. Therefore, it is important that the negotiations happen early in the implementation process and role assignments are periodically revisited.

Our findings also highlight that attitudes about rWGS are influenced by concerns about future availability and potential adverse effects. Our findings are consistent with and extend the previous literature by suggesting that the current expense and lack of payor coverage for first-tier testing may deter individuals from engaging in genomics implementation [34]. As the coverage issue is addressed, such as through the recently announced Medicaid rWGS policy in Michigan [35], it will be interesting to see if there is an increased investment in learning about genomic testing by frontline clinical staff, and how attitudes toward implementation are affected. Concerns about potential future adverse effects for patients and families may also be addressed through education as well as through policy advocacy to protect patient rights about future genomic data usage.

Our findings should be considered in context of several limitations and strengths. First, there may be selection bias because recruitment occurred through local clinical champions, and hospitals that more recently joined the project were under-represented in the sample. Second, perceived knowledge may not reflect actual knowledge level. Third, we did not have detailed information about the implementation strategies employed at each site and this, among other unmeasured factors, may have influenced knowledge and attitudes about rWGS. Strengths of the study included the relatively large sample, multiple hospitals and diverse characteristics of the sites. Along with the inclusion of diverse health professionals from multiple units and departments within the hospitals, these design features support the generalizability of the findings.

## 5. Conclusions

In summary, pediatric healthcare professionals’ excitement to implement genomics is tempered by need for more knowledge and reimbursement as well as concerns about future use of genomic data that could lead to adverse impact on patients and families. Wider education and ongoing implementation research about all aspects of rWGS, from test information, insurer coverage, workflows, to bedside discussions with families, are needed such that wise deployment and full potential of rWGS can be realized.

## Figures and Tables

**Table 1:** Respondent characteristics (N = 307).

Characteristic (*n*)	Mean (SD) or *n* (%)
Age in years (*n* = 302)	42.12 (12.77)
Years in practice (*n* = 304)	15.25 (12.77)
Gender (*n* = 305)	
Female	251 (82%)
Race (*n* = 298)	
White	262 (88%)
South Asian	18 (6%)
African American	8 (3%)
Hispanic	5 (2%)
Multiracial/Other	4 (1%)
Primary position	
Physician—attending	80 (26%)
Physician—resident	26 (9%)
Nurse practitioner	19 (6%)
Genetic counselor	13 (4%)
Nurse (direct patient care)	130 (42%)
Pharmacist, Therapist, Social worker, Parent liaison	12 (4%)
Laboratory director	3 (1%)
Laboratory staff	6 (2%)
Hospital administrator	8 (3%)
Nursing leader ^1^	10 (3%)
Attending physician (*n* = 76)	
Neonatologist/ Intensivist	31 (41%)
Pediatrician/ Hospitalist	19 (25%)
Pediatric subspecialty ^2^	14 (18%)
Geneticist	12 (16%)
Primary Unit (*n* = 304)	
NICU	141 (46%)
Multiple units/hospital wide	58 (19%)
PICU	37 (12%)
Medical surgical	21 (9%)
Outpatient clinic	27 (7%)
Non-clinical	14 (5%)
Laboratory	3 (1%)
Emergency room	3 (1%)

^1^ Nursing leader: nurse director, nurse manager, clinical nurse specialist, case manager; ^2^ Pediatric subspecialty: cardiology, neurology, immunology, oncology.

**Table 2:** Genetics or genomics education of respondents.

Type of Education	*n* (%)
On-the-job training	164 (53)
Genetics course in initial professional training	130 (42)
Hospital supported training	112 (37)
Self-directed education (journal articles, etc.)	97 (32)
Continuing education courses in genetics	79 (26)
Genetics course in graduate school	62 (20)
Seminar/workshops in genetics	44 (14)
Genetics conferences	37 (12)
Advanced training in genetics	20 (7)
No specific training	76 (25)

**Table 3:** Factor loadings based on a principal components analysis with oblimin rotation for 18 items regarding rWGS attitudes.

Item	Factor 1: Personal Capability	Factor 2: Potential/ Intention	Factor 3: Implementation
I am confident about interpreting rWGS test reports	**0.935**	−0.154	−0.0602
I am confident about integrating rWGS test results in the care of patients and their families	**0.768**	0.125	−0.075
I am confident in how to access resources to help me if I have questions or concerns about rWGS results	**0.72**	0.129	−0.00583
I can find reliable sources of the information about rWGS when I need it	**0.611**	−0.092	0.27
Using rWGS fits within the processes that I already use to care for my patients and their families	**0.452**	0.147	0.211
My training has prepared me to care for patients and their families at high risk for medical conditions that have a genetic basis	**0.428**	0.212	0.0334
The information generated by rWGS is important for patient care	−0.243	**0.806**	0.178
I believe that rWGS is relevant to my current practice	0.141	**0.748**	−0.0343
Within the next five years, precision medicine based on rWGS will improve clinical outcomes for infants and children	−0.0144	**0.739**	0.032
I would support rWGS testing for appropriate patients if it was widely available in my clinical practice	−0.0109	**0.684**	−0.00894
rWGS will improve my ability to care for patients and their families	0.189	**0.676**	−0.0253
Parents of my patients will be interested in having rWGS for their child	0.291	**0.506**	−0.136
The implementation leaders/team have the necessary qualities and skills to successfully incorporate rWGS into my unit/ hospital’s clinical practice	−0.188	0.231	**0.763**
Leaders have openly endorsed and supported rWGS in visible ways	0.0379	0.00119	**0.75**
A variety of strategies are being used to enable staff to use rWGS to care for patients in my unit/hospital	0.277	−0.132	**0.611**
A clearly designated person or team is leading the effort to incorporate rWGS into my unit/hospital’s clinical practice	−0.213	0.336	**0.585**
Staff have enough time to facilitate the integration of rWGS into clinical practice	0.204	−0.118	**0.584**
Clear goals have been established for integrating rWGS into clinical practice	0.399	−0.117	**0.527**

**Bold** indicates items retained for each factor in the three-factor solution.

**Table 4:** Final regression model predicting factors and total rWGS attitudes score (N = 307). Coefficient (95% CI) (*p* value).

Term	Factor 1: Personal Capability	Factor 2: Potential/Intention	Factor 3: Implementation	Total Attitudes Score
Self-rated level of knowledge about rWGS	0.505 (0.415, 0.596) **(>0.001)**	0.44 (0.357, 0.523) **(>0.001)**	0.378 (0.273, 0.483) **(>0.001)**	5.62 (4.58, 6.66) **(>0.001)**
Clinical role (vs. non-clinical)			0.326 (0.114, 0.538) **(0.0028)**	3.7 (1.62, 5.78) **(>0.001)**
Confidence about future insurance coverage for rWGS	0.219 (0.112, 0.326) **(>0.001)**	0.223 (0.119, 0.326) **(>0.001)**	0.208 (0.0928, 0.324) **(>0.001)**	3.01 (1.87, 4.15) **(>0.001)**
Concerns about potential long-term effects of genomic testing on patients/ families		−0.266 (0.174, 0.358) **(>0.001)**		−1.95 (0.934, 2.97) **(>0.001)**
NICU/PICU (vs. other unit)	−0.285 (−0.464, −0.107) **(0.0019)**			
Concern about racial disparities in use of genomic testing		−0.265 (0.167, 0.363) **(>0.001)**		
Site2 (ref = Site 1)			0.419 (0.216, 0.622) **(>0.001)**	
Adjusted R^2^	0.43	0.44	0.32	0.51

**Bold** indicates *p* < 0.01.

**Table 5:** Themes and illustrative quotes about rWGS implementation and wider adoption.

Themes/Subthemes	Quotes
**Enthusiasm for rWGS in current or future patient care**	
Potential to improve diagnostic accuracy and speed, leading to more effective treatment	“This tool will change how we care for patients; it can offer treatments and early diagnosis to conditions that would have otherwise taken a very long time and additional costs.” (Physician) “Very important to help families get answers sooner and help determine if there are any treatments available that may help.” (Genetic Counselor) “There are definitely some patients who could benefit, especially those whose clinical conditions are hard to explain for other reasons.” (Direct care nurse)
Qualified enthusiasm	“Hoping this test becomes a covered benefit from insurers so no child in need has to go without access.” (Physician) “Cost, resources and utility of results in the near term are not yet certain although expect this to be more obvious in the future.” (Hospital administrator)
**Implementation of rWGS**	
Satisfaction with implementation	“It is well run with our clinical champions coordinating and providing the counseling and education.” (Physician)
Limited experience; interest in learning more	“It has been so great to be able to do rWGS but more education needed for staff and provider understanding/comfort.” (Nurse Practitioner) “I have heard it mentioned while on multidisciplinary rounds, but as a resident, I feel disconnected and not sure where to turn to (aside from our Geneticist) on a day-to-day basis for rWGS testing and information. I do not feel confident in talking about rWGS alone with my patients and would appreciate more education to help this project take flight at my institution.” (Resident)
Role of genetics service	“Our genetic team has been a key for this project and we rely on them.” (Physician) “Until now this has been monopolized by the genetics department. I feel that this should be a more widely accessible test and based on results or concerns then genetics can be contacted. This reminds me of not ordering an echo till a patient is seen by cardiology. The reality is we order echo and work-ups including troponins and BNP etc., then contact cardiology.” (Physician)
**Concerns about wider use of rWGS/genomic testing**	
	“I think case specific rWGS has merit, but the extraneous data that may impact insurability must be non-discoverable by insurance companies, and extreme caution should be used by providers to be sure patients are not overwhelmed by unexpected information.” (Physician) “I feel this is an option for parents but also feel it is a decision they need to make after all the appropriate education is given to them.” (Nurse Practitioner)

## Data Availability

Deidentified data will be shared upon reasonable request directed to Linda S. Franck (linda.franck@ucsf.edu) from qualified investigators beginning 6 months and ending 5 years after study publication.

## Supplementary Materials

The following supporting information can be downloaded at: https://www.mdpi.com/article/10.3390/children9030357/s1, Table S1: Univariate regression models predicting the three factors and total rWGS attitudes score.
