# Supplementary material for: Healthcare Professionals’ Attitudes toward Rapid Whole Genome Sequencing in Pediatric Acute Care
Source: Children (Basel). 2022 Mar 4;9(3):357. doi: 10.3390/children9030357 (PMC8947383; doi:10.3390/children9030357)
Supplement: Supplementary file 1 [file children-09-00357-s001.zip › children-1574927-supplementary.pdf]

Table S1. Univariate regression models predicting the three factors and total rWGS attitudes score (N=307). Coefficient (95% CI) [p-value].

Term	Factor 1: Personal capability	Factor 2: Potential/ intention	Factor 3: Implementation	Total Attitudes Score
Years in role	-0.00152 (-0.0102, 0.00712) [0.73]	-0.00392 (-0.0126, 0.00471) [0.37]	-0.00336 (-0.0118, 0.0051) [0.43]	-0.0359 (-0.133, 0.0615) [0.47]
Age	0.004 (-0.00458, 0.0126) [0.36]	-0.00122 (-0.00982, 0.00738) [0.78]	0.000958 (-0.00751, 0.00943) [0.82]	0.0241 (-0.073, 0.121) [0.63]
Male gender	-0.497 (-0.781, -0.212) [<0.001]	-0.253 (-0.536, 0.0289) [0.078]	-0.488 (-0.762, -0.214) [<0.001]	-5.78 (-8.96, -2.59) [<0.001]
Racial/Ethnic Identity (ref = White)	0.216 (-0.123, 0.555) [0.21]	-0.0196 (-0.351, 0.312) [0.91]	0.156 (-0.175, 0.486) [0.35]	1.74 (-2.06, 5.54) [0.37]
Site2 (ref = Site 1)	0.00407 (-0.241, 0.249) [0.97]	0.0718 (-0.172, 0.316) [0.56]	0.279 (0.0369, 0.522) [0.024]	1.41 (-1.37, 4.2) [0.32]
Other sites (ref = Site 1)	0.401 (0.117, 0.684) [<0.01]	0.327 (0.0416, 0.612) [0.025]	0.174 (-0.105, 0.453) [0.22]	4.04 (0.834, 7.25) [0.014]
NICU/PICU (vs another unit)	-0.618 (-0.829, -0.408) [<0.001]	-0.228 (-0.445, -0.0107) [0.04]	-0.446 (-0.656, -0.235) [<0.001]	-6.12 (-8.52, -3.73) [<0.001]
Clinical role (vs non-clinical)	0.799 (0.597, 1) [<0.001]	0.57 (0.361, 0.778) [<0.001]	0.65 (0.448, 0.852) [<0.001]	9.3 (7.04, 11.6) [<0.001]
No genetics/genomics training	-0.799 (-1.04, -0.553) [<0.001]	-0.677 (-0.924, -0.43) [<0.001]	-0.653 (-0.896, -0.411) [<0.001]	-9.68 (-12.4, -6.95) [<0.001]
Self-rated level of knowledge about rWGS	0.581 (0.494, 0.669) [<0.001]	0.486 (0.392, 0.58) [<0.001]	0.46 (0.367, 0.554) [<0.001]	6.95 (5.99, 7.91) [<0.001]
Concerns about potential long-term effects of genomic testing on patients/families	-0.0412 (-0.0845, 0.167) [0.52]	-0.276 (0.157, 0.395) [<0.001]	-0.0534 (-0.0748, 0.182) [0.41]	-1.49 (0.0595, 2.92) [0.041]
Confidence about future insurance coverage for rWGS	0.344 (0.211, 0.476) [<0.001]	0.371 (0.242, 0.499) [<0.001]	0.295 (0.161, 0.43) [<0.001]	4.49 (2.99, 5.99) [<0.001]
Concern rWGS will lead to insurance discrimination	-0.14 (-0.27, -0.0104) [0.034]	-0.078 (-0.206, 0.0502) [0.23]	-0.0545 (-0.186, 0.0774) [0.42]	-1.27 (-2.75, 0.216) [0.094]
Concern about racial disparities in use of genomic testing	-0.106 (-0.0253, 0.237) [0.11]	-0.37 (0.248, 0.491) [<0.001]	-0.0978 (-0.0342, 0.23) [0.15]	-2.37 (0.894, 3.84) [0.0017]

Considers patient's race/ancestry in disease risk and treatment decisions	0.00381 (-0.122, 0.13) [0.95]	0.163 (0.0397, 0.286) [0.0097]	-0.0163 (-0.145, 0.112) [0.8]	0.619 (-0.815, 2.05) [0.4]
Conversations with families about rWGS testing or diagnosed disorder (past 6 months)	0.84 (0.607, 1.07) [<0.001]	0.619 (0.381, 0.857) [<0.001]	0.52 (0.282, 0.757) [<0.001]	9.05 (6.4, 11.7) [<0.001]
Involved in the care of an inpatient child for whom rWGS was ordered (past 6 months)	0.466 (0.25, 0.681) [<0.001]	0.697 (0.492, 0.902) [<0.001]	0.597 (0.392, 0.803) [<0.001]	7.68 (5.33, 10) [<0.001]
Cared for patients/families referred for genetic counseling as a result of rWGS testing (past 6 months)	0.475 (0.259, 0.69) [<0.001]	0.659 (0.454, 0.864) [<0.001]	0.486 (0.276, 0.696) [<0.001]	7.12 (4.75, 9.49) [<0.001]

rWGS: rapid whole genome sequencing
